# Advancements in Preprocessing and Analysis of Nitrite and Nitrate since 2010 in Biological Samples: A Review

**DOI:** 10.3390/molecules28207122

**Published:** 2023-10-17

**Authors:** Guojie Liu, Honghui Guo, Wanlin Zhao, Hongmu Yan, Enze Zhang, Lina Gao

**Affiliations:** 1 Department of Chemistry, School of Forensic Medicine, China Medical University, Shenyang 110122, China; gjliu@cmu.edu.cn; 2 Liaoning Province Key Laboratory of Forensic Bio-Evidence Sciences, Shenyang 110122, China; 3 Center of Forensic Investigation, China Medical University, Shenyang 110122, China; 4 Forensic Analytical Toxicology Department, School of Forensic Medicine, China Medical University, Shenyang 110122, China; 5 First Clinical College, China Medical University, Shenyang 110122, China

**Keywords:** nitrite, biological samples, nitrate, analytical methods, sample treatment

## Abstract

As a substance present in organisms, nitrite is a metabolite of nitric oxide and can also be ingested. Nitrate is the metabolite of nitrite. Therefore, it is necessary to measure it quickly, easily and accurately to evaluate the health status of humans. Although there have been several reviews on analytical methods for non-biological samples, there have been no reviews focused on both sample preparation and analytical methods for biological samples. First, rapid and accurate nitrite measurement has significant effects on human health. Second, the detection of nitrite in biological samples is problematic due to its very low concentration and matrix interferences. Therefore, the pretreatment plus measuring methods for nitrite and nitrate obtained from biological samples since 2010 are summarized in the present review, and their prospects for the future are proposed. The treatment methods include liquid–liquid microextraction, various derivatization reactions, liquid–liquid extraction, protein precipitation, solid phase extraction, and cloud point extraction. Analytical methods include spectroscopic methods, paper-based analytical devices, ion chromatography, liquid chromatography, gas chromatography–mass spectrometry, electrochemical methods, liquid chromatography–mass spectrometry and capillary electrophoresis. Derivatization reagents with rapid quantitative reactions and advanced extraction methods with high enrichment efficiency are also included. Nitrate and nitrate should be determined at the same time by the same analytical method. In addition, much exploration has been performed on formulating fast testing through microfluidic technology. In this review, the newest developments in nitrite and nitrate processing are a focus in addition to novel techniques employed in such analyses.

## 1. Introduction

Nitrite has extensive applications as a food additive for meat preservation. A moderate amount of nitrite has bacteriostatic and preservative effects, but excessive or long-term intake of nitrite can lead to various diseases, such as circulatory system failure, cancer, and teratogenicity [1,2,3,4]. In the field of forensic medicine, there are many cases of nitrite poisoning, as nitrite penetrates the bloodstream and can stimulate hemoglobin leading to irreversible conversion to methemoglobin, thereby impairing oxygen uptake and delivery [2,4]. On the other hand, nitrogen oxide (NO), as a new type of cell messenger molecule, has a role in numerous physiological and pathological processes in the body, and has received wide attention from the medical community over the past few years [4,5]. The determination of nitrate and nitrite content can indirectly reflect the level of NO as NO can be quickly converted into nitrate as well as nitrite due to its extremely short half-life and in vivo chemical instability [6,7,8]. Unfortunately, there is no simple and feasible means of analyzing nitrate and nitrite obtained from biological samples. In particular, nitrite concentration in the human body is very low, approximately one hundred times lower than that of nitrate, while nitrite toxicity is several hundred times that of nitrate [2]. Consequently, a rapid, simple, specific and responsive method is urgently required to measure nitrite and nitrate in biological samples to predict the health of humans or monitor nitrite poisoning.

However, many researchers have reviewed the methods for the determination of nitrite in recent years, such as Moorcroft and colleagues in 2001 [7], who reviewed nitrate and nitrite detection mainly acquired from environmental samples. In 2005, Tsikas et al. [8] summarized various quantitative approaches to examine nitrate and nitrite as metabolic products of NO in human biological fluids. In 2007, Jobgen et al. [9] described different high-performance liquid chromatography (HPLC) methods used to examine nitrate and nitrite in biological samples. Wu et al. (2013) [10] reviewed the methods to determine NO-derived nitrate and nitrite ions obtained from biological samples. In 2017, Wang et al. [4] updated the methods used for determination of nitrate and nitrite. All these reviews mainly focused on assays for nitrate together with nitrite. Wierzbicka et al. (2020) summarized the detection methods for nitrate and nitrite in water systems, sewage, as well as in food [11]. The sample pretreatment and determination methods should be chosen according to the matrices present. Thus, analytical methods suitable for both environmental and food matrices may not necessarily be applicable for measuring nitrate and nitrite in biological samples. Given that nitrite and nitrate have low concentrations and high water solubility, selecting suitable extraction and clean-up procedures to examine these analytes in biological samples is important. Most reviews have focused on measurement methods used primarily for non-biological samples.

With this background, an up-to-date and comprehensive review is urgently needed. In this article, we summarize the pretreatment and determination methods for nitrite and nitrate since 2010. Moreover, we compare various sample pretreatment methods and various detection methods. In addition, we express our views on future development trends. The objective of this article is to supply more comprehensive information about the pretreatment and determination methods for nitrite and nitrate in biological samples for analytical chemist.

## 2. Sample Pretreatment

Sample pretreatment for accurate nitrite and nitroxide measurement in biological specimens is important for three reasons. First, the biological sample matrix is complex. Second, biological specimens have low NO and nitrite content. Third, nitrite and NO are unstable, and are often metabolized into other forms. Therefore, appropriate sample pretreatment methods are needed to ensure the sensitivity of the subsequent determination. Simple sample pretreatment technologies [12,13], liquid–liquid extraction (LLE) [14,15,16,17], cloud point extraction (CPE) [18,19,20],liquid phase microextraction (LPME) [21,22,23,24,25], on-line solid phase extraction (SPE) [26], solid phase microextraction (SPME) [27], aqueous two-phase extraction (ATPE) [28], and electromembrane extraction (EME) [29] are summarized in Table 1.

### 2.1. Simple Sample Treatment

Biological samples have many impurities affecting nitrate and nitrite determination, such as minerals, proteins, urea, fatty acids, ammonia, sugars, biological amines, and amino acids [10]. Moreover, macromolecules, such as enzymes, soluble proteins, lipids, and hemoglobin, are usually found in the serum and urine, and can interfere with reactions such as derivatization and reduction. In addition, other substances can cause colorimetry interference. Nitrite and nitrate are usually found in biological samples such as urine and saliva, matrices of which have less interference than blood. The dilution method is used for pretreatment of urine and saliva. Whole blood and red blood cells interfere with the determination of nitrite because they contain hemoglobin, whereas serum and plasma without hemoglobin do not interfere with nitrate and nitrite determination. However, in forensic science, whole blood and red blood cells should not be the first choice for nitrite detection. Protein precipitation for blood samples and decolorization for urine samples are the basic means of sample preparation. For protein precipitation, researchers may use alkaline solution and salt solutions such as NaOH and ZnSO_4_, as well as acetonitrile, methanol, and other organic solvents; or ultrafiltration methods [12,18,19]. Ultrafiltration, a frequently used method for eliminating large molecules such as proteins, may fail to screen out certain low molecular weight compounds including glucose and amino acids.

### 2.2. Evolution of Liquid–Liquid Extraction

The LLE method has found widespread application as a trace enrichment approach in analytical chemistry. However, conventional LLE has clear disadvantages, such as a need for abundant organic solvents with high purity and toxicity, a low enrichment factor (EF), time-consuming operation, and environmental pollution [14,15,16,17]. To overcome these problems and adapt to the development of modern analytical technology, CPE, LPME, and ATPE have emerged in recent years, thus addressing the disadvantages of LLE.

CPE, an emerging LLE technology using a surfactant to extract the analyte from samples, with little or no organic solvent, is an environmentally friendly sample pretreatment method [19,20].

**Figure 1:**
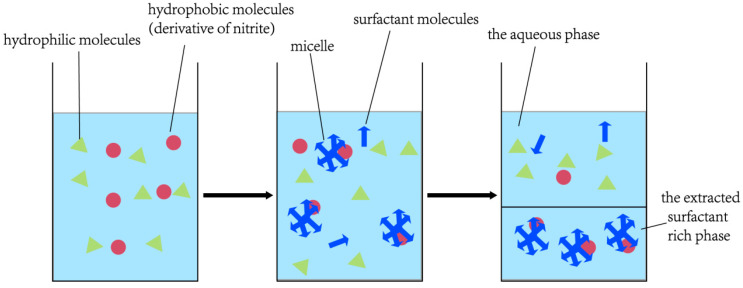
The process of CPE.

Pourreza et al. [20] reported some key parameters which had an impact on CPE extraction efficiency, such as the amount of Triton X-100, equilibrium time, as well as the influence of incubation time and equilibration temperature, with Triton X-100 as a CPE solvent. The optimal conditions have been found to be 8 mL of Triton X-100, a 30 min equilibrium time, an 80 °C equilibrium temperature, and a 25 min incubation time. This method is used for water and sausage samples, and recoveries of 91.2−103.0% are obtained. An approach on the basis of the Griess reaction and CPE technique was explored by Zhao et al. [19], which was adopted to examine blood and urine samples for nitrite content with non-ionic surfactant Triton-X114 as an extraction solvent, yielding a recovery ranging from 92.6% to 101.2%. The process of CPE is given in Figure 1.

Another LLE mode, ATPE, utilizes aqueous solutions with completely different natures in both liquid phases for the purpose of realizing phase separation. It has the advantages of fast processing speed and no inactivation or denaturation of bioactive substances. Liu et al. [28] used tetrahydrofuran as the extraction solvent in ATPE to extract the derivatization of nitrite and then analyze nitrite in urine samples by spectrofluorimetric determination, thus obtaining recoveries of 93.3–105.0%.

### 2.3. Liquid Phase Microextraction

LPME, used as an innovative sample preprocessing technique in the 1990s, does not have the drawbacks of traditional LLE, including cumbersome operation, use of large amounts of organic solvents, and environmental pollution. LPME has the advantages of low solvent consumption (only microliter level), high extraction efficiency, and easy operation and automation [23,25,38,39].

**Figure 2:**
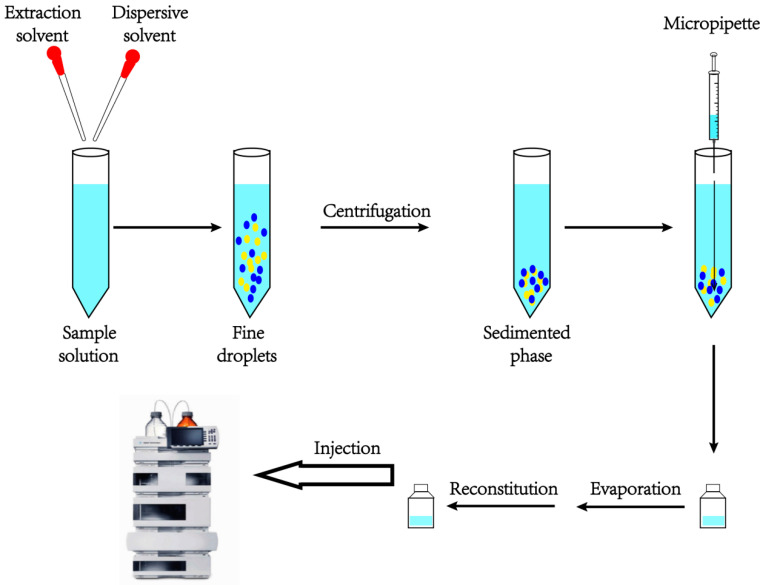
The common procedure of DLLME.

Dispersion liquid–liquid microextraction (DLLME), a newer microextraction technology based on LLE, ref. [21], was first developed by Rezaee and colleagues in 2006. The extraction system consists of three parts: extraction solvent, dispersion solvent and sample solution. The ability of the dispersing solvent is miscibility with the other two components, dispersing the extraction solvent to very fine droplets, dispersing into the water phase. Figure 2 shows the common procedure of DLLME.

Senra-Ferreiro et al. [23] detected nitrite in water samples via headspace single drop liquid phase microextraction (HS-SDME) plus micro-spectrophotometry, and investigated several key parameters such as acetic acid concentration, extractant phase volume and composition, sample volume, ionic intensity, sample agitation, temperature and microextraction times. Under optimized conditions, a limit of detection (LOD) of 1.5 μg/L and an EF of 193 were obtained.

Ionic liquid (IL) as a “green chemical” solvent is replacing the toxic, combustible and volatile organic solvents of traditional LLE [40,41]. Owing to its advantages such as simple operation, rapidity, high enrichment efficiency, and diminished use of extraction agent, ionic liquid-dispersive liquid–liquid microextraction (IL-DLLME) is applicable for combined use with instrumental methods covering gas chromatography and liquid chromatography [42]. He et al. [24] utilized the ionic liquid 1-octyl-3-methylimidazolium bis[(trifluoromethyl)sulfonyl]imide ([OMIM][Tf_2_N]) as an extraction solvent in combination with a HPLC-UV detector to analyze nitrite in saliva, and obtained a good EF (413) and extraction recovery (ER) of 94.2%.

A hydrophobic deep eutectic solvent is similar to IL in terms of physical properties and phase behavior, which can serve as a novel green extraction solvent to substitute IL. Deep eutectic solvents were composed of hydrogen bond acceptor and hydrogen bond donor at a certain mole ratio [25,43]. Zhang et al. [25] used deep eutectic solvents-based DLLME (DESs-DLLME) plus HPLC to detect nitrite levels in saliva and urine samples from humans, and successfully used this method to measure nitrite in three environmental water specimens and two biological samples, with recoveries ranging from 90.5% to 115.2%.

**Figure 3:**
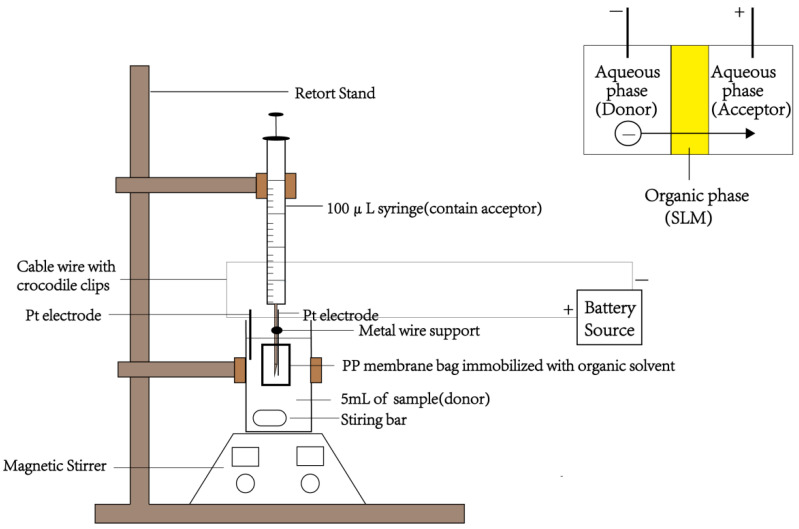
Schematic of EME. Reproduced from ref. [29].

EME is a novel sample pretreatment technology representing a new development in combining liquid phase microextraction with an electric field. Tan et al. [29] pioneered EME coupled with ion chromatography to examine amniotic fluids for nitrite level determination. EME performed more efficiently than HF-LPME, exhibiting a higher EF (6.4) and shorter extraction time (5 min). Figure 3 shows the procedure of EME reported by Tan et al. [29].

### 2.4. SPE plus SPME

SPE, compared with traditional LLE, markedly improves isolation and purification efficiency. Traditional SPE procedure usually contains four steps: precondition of cartridges, sample loading, washing and elution of analytes. Notably, on-line SPE is labor-saving and time-saving, and thus is commonly applied in batch sample analysis. Hu et al. [26] employed a C18 SPE column to separate the analyte after derivatization with 2,3-diaminonaphthalene, where the entire run time for the on-line SPE was 10 min. Furthermore, satisfactory recovery (99–112%) indicated its great potential in nitrite and nitrate detection.

The development of SPME is another direction in SPE. SPME offers numerous advantages (e.g., high enrichment capacity and accuracy). Yang et al. [27] employed HS-SPME to extract nitrite from samples based on a 100 μm polydimethylsiloxane (PDMS) absorbent. HS-SPME showed good sensitivity (0.1 μg/L) and reproducibility (96–103%).

### 2.5. Summary

Nitrite concentrations are about one hundred time lower than those of nitrate, while its toxicity is about the same order of magnitude higher (LD_50_ was 85 mg/kg for nitrite, and 3236 mg/kg for nitrate (rat oral)) [31]. Given nitrite’s unstable nature and possible metabolism to nitrate, and the presence of complex biological sample matrices, highly efficient pretreatment technology is necessary for the detection of nitrite in biological samples. For simple matrices, such as urine and saliva, samples can be diluted or treated by ultrafiltration. For blood samples rich in hemoglobin, an organic solvent or salt is generally added for protein precipitation, and membrane filtration is subsequently used for final clean-up. If ion chromatography (IC) is used as an analytical technique, an Ag cartridge and Na cartridge can be used to remove the interference of Cl^−^. However, efficient extraction methods such as CPE are based on traditional LLE and their advantages include higher EFs and environmental friendliness. In recent years, IL-DLLME and DESs-DLLME have achieved higher EF, thus aiding in the determination of trace nitrate. For bulk sample analysis, automated preprocessing techniques, such as on-line SPE, are needed. In the future, simple, high-efficiency sample pretreatment is expected to remain a focus for researchers. In Table 2, these sample pretreatments are compared, and their advantages and disadvantages are illustrated.

## 3. Analytical Methods

**Figure 4:**
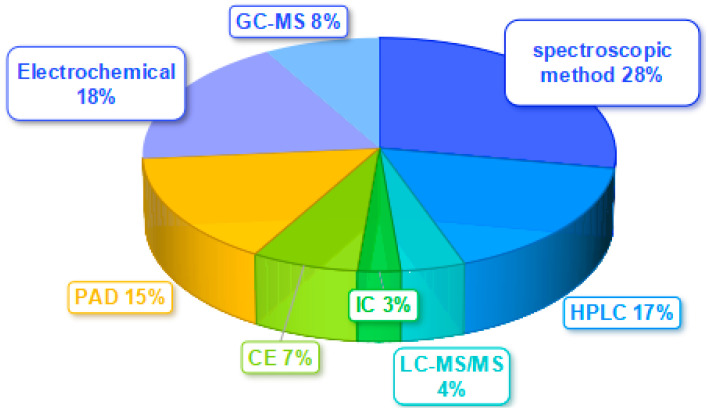
Various analytical methods for assessing nitrate and/or nitrite in biological specimens.

Diverse analytical approaches have been developed in recent decades to measure nitrite and nitrate, including spectroscopic methods (spectrophotometry, chemiluminescence, colorimetry, spectrofluorometry, and Raman spectroscopy), electrochemistry, CE, HPLC, gas chromatography–mass spectrometry (GC-MS), HPLC-MS, IC, and paper-based analytical devices (PAD), which are summarized in Figure 4.

### 3.1. Spectroscopic Methods

Spectrophotometry is widely used due to the universal availability of instruments, simplicity of procedures, and low cost. In addition, it provides high sensitivity when appropriate chromogenic reagents are available. A variety of spectroscopic techniques are used, including spectrophotometry [12,19,27,35,37,44,45], spectrofluorometry [28,46,47,48], chemiluminescence [6,28,49,50], colorimetry [51,52,53,54,55], and Raman spectroscopy [56,57]. Table 3 summarizes the data on nitrite and nitrate detection by spectrophotometry, spectrofluorometry and colorimetry. In general, regardless of simple measurement and a lower detection limit, spectroscopic methods also have several disadvantages: they suffer from interference of other substances as well as certain ions (trisodium citrate, EDTA, etc.) [45,52,53,54].

#### 3.1.1. Spectrophotometry

Spectrophotometry is still the most frequently used method for nitrite measurement. However, some spectrophotometric methods have disadvantages such as insufficient sensitivity or expensive/toxic reagents [12]. Nagaraja et al. [12] developed a spectrophotometric method for nitrite detection, which showed simplicity, sensitivity, fair selectivity and high efficiency, and used AHNDMS diazotized intramolecular coupling through a phosphate buffer solution (PBS) at pH 7.5. Within the 0.1–1.6 μg/mL range, there was a good linear relationship, yielding an LOD of 6.9 ng/mL. The method has been successfully used for human saliva, with recoveries of 98%.

**Figure 5:**
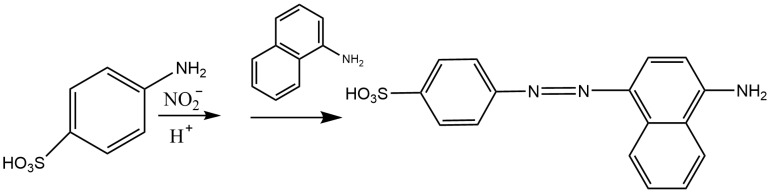
Griess reaction (nitrite reacts with p-aminobenzenesulfonic acid in acid conditions, and then reacts with 1-naphtylamine).

Zhao et al. [19] developed a method with reference to the Griess reaction, whose principle is illustrated in Figure 5. After the reaction, a purple dye that can be detected at 409 nm is formed. Under optimal conditions, nitrite shows good linearity within 10–400 ng/mL (LOD of 2.5 ng/mL). Successful utilization of this method to detect nitrite in blood and urine specimens is achieved with recoveries of 92.6–101.2%.

**Figure 6:**
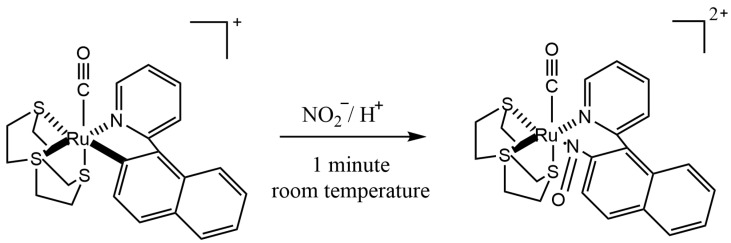
Diazotization of [Ru(npy)([9]aneS_3_)(CO)](ClO_4_) ruthenium complex.

Lo et al. [44] used [Ru(npy)([9]aneS_3_)(CO)](ClO_4_) diazotization, a developed recently ruthenium complex, according to a principle shown in Figure 6. After the addition of acidified NO_2_^−^, the derivatives exhibit maximum absorption at 483 nm. The method has good linearity (over the range of 1–840 µmol/L) and good sensitivity (0.39 µmol/L).

#### 3.1.2. Spectrofluorometry

Unnikrishnan et al. [46] formulated a nitrite spectrofluorimetric method with the following principle: in the core of bovine serum albumin (BSA)-Au25 nanoclusters (NCs), nitrite ion triggers Au(0) atom oxidation into Au(I) atoms which induces fluorescence quenching. Given the higher biocompatibility and proneness to bioconjugation, BSA-Au NCs can be widely used to determine small molecules and metal ions. The LOD with this method is 50 nM. However, the method is also prone to some interference from inorganic ions, including CN^−^ and S^2−^.

Ning et al. [48] reported an “on–off–on” fluorescence method for determination of nitrite. The principle of this method is as follows. First, near-infrared carbon dots (NIR-CDs) doped with fluorine and nitrogen are constructed. Second, a red BPS-Fe^2+^ complex is added for NIR-CDs fluorescence quenching. Finally, after adding nitrite to the acidic environment, nitrite oxidizes Fe^2+^ to produce Fe^3+^, thereby resulting in fluorescence recovery of NIR-CDs. Good linearity within 1−50 μM together with an LOD of 0.056 μM are achieved with this method.

Graphene quantum dots (GQDs) are an appropriate choice of fluorescent materials, as they have many unique properties such as robust chemical inertness, abundant availability, photo-bleaching resistance, outstanding water solubility and biocompatibility, and low cytotoxicity, resulting in them being widely applied to the exploration of fluorescence sensors with high selectivity and sensitivity over the past few years. Jin et al. [47] developed a spectrofluorimetric method by virtue of GQD fluorescence quenching, which is used for nitrite detection in urine specimens. A breakthrough in sensitivity is achieved with this method, with an LOD of 5.4 nmol/L.

#### 3.1.3. Colorimetry

Enormous interest is attached to colorimetric detection due to its feasibility of naked-eye observation of results in addition to its simplicity [51,52,53,54,55].

Siu et al. [51] used an instantaneous quantitative colorimeter to measure nitrite in urine specimens, producing preferable linearity in the salt buffer over the 0.78−200 μM range. Artificial urine shows an LOD of 1.6 μM. The method is advantageous in terms of low cost, portability, and quantitation of nitrite in urine, and can be applied in at-home health monitoring. According to Zhang et al. [55], a fast method based on specific reaction of peroxynitrous acid (HOONO) was developed and used to generate a golden yellow product through oxidization of colorless 3,3′,5,5′-tetramethylbenzidine (TMB). An LOD of 100 nM was obtained with this method. However, this colorimetric method lacks sensitivity.

#### 3.1.4. Chemiluminescence

**Figure 7:**
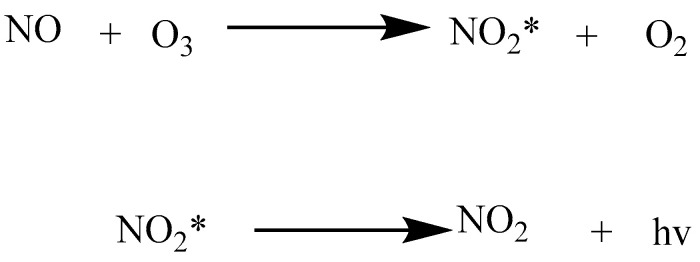
The chemiluminescence reaction of nitric oxide with ozone (* Represents an excited state).

As chemiluminescence bound to triiodide reducing solution allows accurate quantification of nitrite in a low nanomolar concentration range, it is deemed a more responsive method [49]. Depending on the chemiluminescence reaction with ozone, NO was derived from nitrate and nitrite through reduction [6,42,43]. The specific reaction is shown in Figure 7.

This method considers the influence of other nitrogen oxides on the reaction, and the determination steps in this method are briefly described as follows.

First, the sample is divided into two parts. One part is treated with acid sulfonamide for nitrite diazotization, which cannot release NO, followed by the addition of triiodide reducing agent (I_3_ solution) which allows the release of NO by the remainder of the nitrogen oxide (including nitrate), then, finally, a reaction with O_3_. A nitrogen oxygen analyzer is used for detection. Second, using the remaining part, a reducing agent is added to reduce all the nitrogen oxide and release NO, which is then followed by a reaction with O_3_, and a nitrogen–oxygen analyzer is used for detection. Finally, the difference between the two results is used to obtain the NO_2_^−^ content.

Although the pretreatment process is complicated, it can accurately determine the NO_2_^−^ content and eliminates the interference by other nitrogen oxides in the process. Piknova et al. [50] investigated the effects of different reducing agents. Nitrite can be selectively determined via ascorbic acid/acetic acid solution, which is specific for nitrite, while I_3_ and a vanadium trichloride (VCl_3_) solution can reduce all nitrogen oxides (nitrite, nitrate, R-SNO, Fe-NO and R-NNO functional groups, etc.) to produce NO, which reacts with ozone. Therefore, for different analytes, we can choose different reduction reagents. If nitrite is only detected, ascorbic acid/acetic acid can be chosen. If the NO content is detected, the I_3_ reduction reagent and VCl_3_ can be chosen. The LOD of this method is 20 nmol. Expensive devices that are only available in highly specialized laboratories are needed for chemiluminescence. Moreover, chemical chromogenic agents are more toxic and harmful to the health of analysts [33,36].

#### 3.1.5. Raman Spectroscopy

Despite its ability to distinguish chemical structure, conventional Raman spectroscopy, a crucial analytical method, has a primary limitation of low sensitivity caused by unfavorable effectiveness of inelastic scattering. Currently, most researchers focus on the substrate; for example, Ag nanoparticles in surface enhanced Raman spectroscopy (SERS).

Zhang et al. [56] developed the shell-isolated nanoparticle-enhanced Raman spectroscopy (SHINERS) to measure nitrite ions in complex samples, characterized by simplicity, speed and selectivity. At the range of 0.5−6.0 mg/L, there was linearity, and an LOD of 0.07 mg/L was obtained. This method has some advantages; for example, there is no requirement for sample pretreatment, the method also displays high selectivity, the entire analysis is completed within 10 min, and few samples are consumed (about μL level). This method is also successfully used for nitrite detection in saliva.

**Figure 8:**
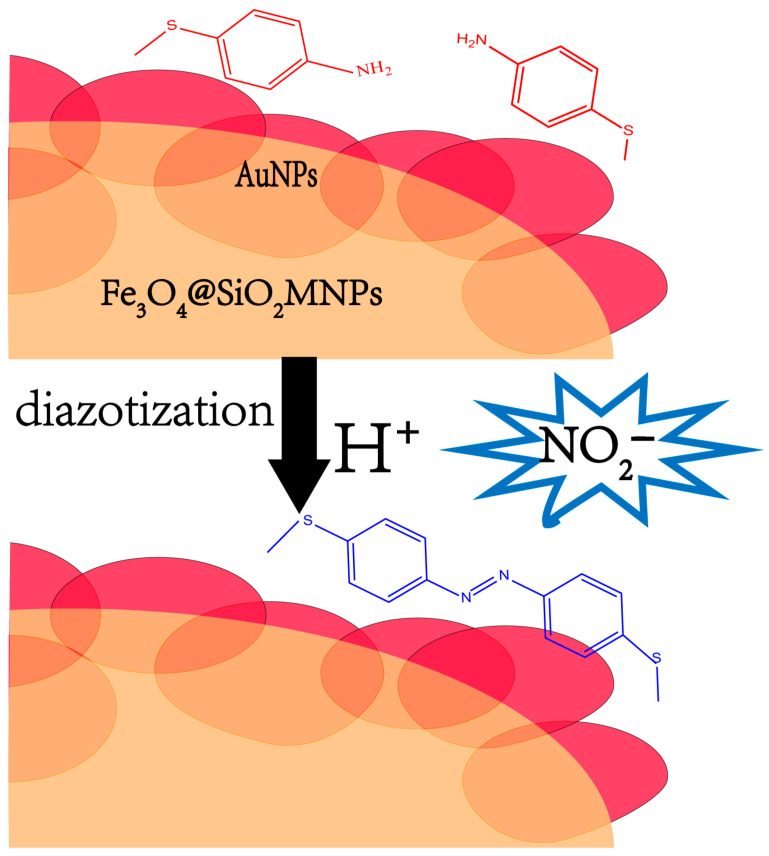
Principle of the nitrite reaction with 4-ATP on Fe_3_O_4_@SiO_2_/Au MNPs.

Chen et al. [57] described a method based on SERS for Fe_3_O_4_@SiO_2_/Au magnetic nanoparticles (MNPs) to detect nitrite combined with derivatization. In acid conditions, conjugation between Fe_3_O_4_@SiO_2_/Au MNPs and 4-aminothiophenol (4-ATP) molecules occurred easily. This method is selective for nitrite, as nitrite can react with 4-ATP for diazotization as shown in Figure 8. Using this method, SERS signals were found to have excellent linearity between 10 μM and 100 μM. This allowed for the detection of three representative peaks, and NO_2_^−^ concentrations as low as 15.63 μM, 13.69 μM, and 17.77 μM, respectively, were obtained. This method has the advantages of simplicity, speed, specificity and no sample pretreatment. The method was also successfully used in water samples and urine, but the sensitivity requires further improvement.

### 3.2. HPLC Methods

**Figure 9:**
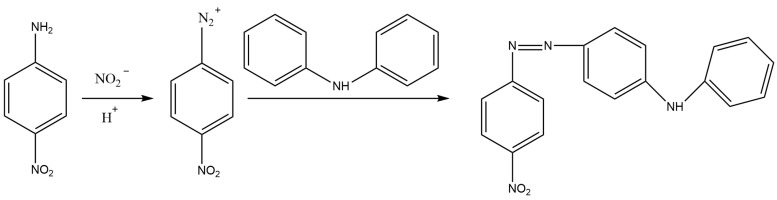
Reaction between *p*-nitroaniline and nitrite ion, with acid media containing diphenylamine.

HPLC systems are available in many laboratories and are typically used for nitrate or nitrite detection in biological specimens. Usually, the Griess reaction in combination with different extraction techniques are adopted [10,18,24,25,31,37,38,58,59,60,61] (Table 4). He et al. [24] developed a method for the reaction between *p*-nitroaniline and nitrite ion, with acid media containing diphenylamine, which was used in combination with HPLC-UV to detect nitrite levels in saliva specimens (the reaction principle is shown in Figure 9). This method was validated as feasible and practical with high repeatability (Relative Standard Deviation (RSD) < 4.1%)and sensitivity (LOD: 0.05 μg/L).

Zhang et al. [25] used DESs-DLLME after the Griess reaction combined with HPLC-UV to detect nitrite in saliva and human urine samples. The method showed a good linear relation within 1–300 μg/L and high sensitivity (LOD: 0.2 μg/L, LOQ: 1 μg/L) under optimized conditions.

Tatarczak-Michalewska et al. [59] performed a method using a phosphatidylcholine column combined with potassium permanganate under acidic conditions as an oxidant to examine nitrate and nitrite content in saliva. Nitrates can be immediately detected via this method by the absorbance at approximately 210 nm. However, nitrite was converted into nitrate through oxidization using potassium permanganate in an acidic environment. The LOD was 4.56 ng/mL (nitrate) and 4.21 ng/mL (nitrite).

**Figure 10:**
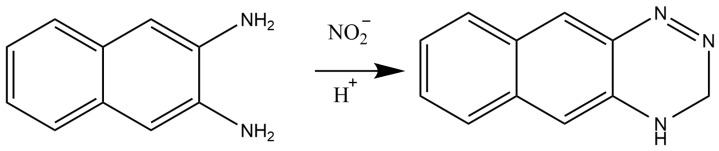
Interaction between DAN and nitrite for NAT production.

Ata et al. [58] described a method where a fluorescence detector was coupled with HPLC. This method showed higher sensitivity for nitrite (LOD: 0.13 ng/L) and nitrate (LOD: 0.19 ng/L). With regard to the fluorometric assay for nitrite, 2,3-naphthotriazole (NAT) was generated on the basis of the reaction between 2,3-diaminonaphthalene (DAN) and nitrite, as shown in Figure 10.

### 3.3. HPLC-MS

As the sole analytical technique for detecting nitrate and nitrite isotopes, MS instruments have witnessed popularization due to their sensitivity, specificity and quantitative accuracy. For biological specimens, LC-MS/MS generally has correlations with matrix effects [62], as the matrix such as sugars and protein affect the accurate quantification to some extent [63]. Human plasma and distilled water are frequently compared for the quantification results of nitrite, for the purpose of assessing the impact of human plasma components on the matrix.

The protein is removed by ultrafiltration, and the nitrite is converted to S-nitrosoglutathione, and then detected by HPLC-MS/MS as reported by Hanff et al. [30]. With a total analysis time of no more than 5 min and peak onset time of 0.8 min, the method shows complete verification using human plasma (range: 0 nM−2000 nM), yielding an LOD of 1 fmol and an LOQ of 5 nM for nitrite. Moreover, the method also has high accuracy due to the supplemented internal standard (IS) isotope marker, and the recovery rate is 91.7−107.6%. The IS significantly enhances the relative matrix effect by nearly 21%.

On-line SPE in combination with LC-MS/MS is utilized to examine nitrite content in urinary and fecal excretion [26] after derivatization with DAN. According to the results, the LOQs declines to 1 nM and 10 nM for 15 NO_2_^−^ and 15 NO_3_^−^, respectively. The urine and feces specimens in this study exhibit matrix effects of −2.3–9.5% and 0–8.0%, respectively.

### 3.4. Ion Chromatography

Compared with other instruments, ion chromatography (IC) has great advantages in terms of precision, accuracy and other aspects in nitrite detection. Following the introduction of an elution automatic generator, the required elution concentration can be generated online using deionized water, avoiding the error caused by manual configuration. While simplifying the test procedure, it improves the reproducibility of the test results and reduces environmental pollution.

The overall running time of IC is 45 min as reported by Yan et al. [32], and the nitrite retention time is 13.8 min. The sample pretreatment process requires a guard cartridge, Ag cartridge and Na cartridge. The LOD in this method is 0.4 μmol/L, and the LOQ is 2 μmol/L in whole blood.

Kim et al. [64] detected nitrite in whole blood samples by means of a conductivity detector together with IC. Samples were prepared using only a simple ultrafiltration method. Given the interference in nitrite during IC methods under general conditions, chloride ions were thoroughly separated using a potassium hydroxide (KOH) gradient elution. Nitrite showed an LOD of 0.5 mg/L and LOQ of 1 mg/L. At 1−500 mg/L and 5−500 mg/L of nitrite and nitrate, respectively, favorable linearity was achieved in both cases.

### 3.5. Capillary Electrophoresis

Capillary electrophoresis (CE) has been recognized as a powerful analytical tool for nitrite and nitrate in biological samples because of its high sensitivity, superior separating effectiveness, and suitability for isolating charged samples. CE also has advantages of low sample consumption, inexpensive experimental reagents, simple operation, and a broad application range.

Capillary zone electrophoresis (CZE) approaches have been used with a direct UV-–Vis detector for examining nitrate and nitrite levels in biological fluids at 214 nm [33]. The main contributing factor for the CZE mode is related to complex background electrolytes (BGE), electroosmotic flow (EOF) reverse additives, and instability of EOF (varied with pH value). These issues may lead to relatively unsatisfactory reproducibility and accuracy.

The optimized parameters include separation voltage, capillary temperature, and the effect of injection time when nitrate and nitrite are measured by CZE. Wang et al. [33] used human plasma to detect nitrate and nitrite through field-amplified sample stacking (FASS) in combination with on-capillary preconcentration. Estimated LODs (0.07 mM and 0.05 mM) and LOQs (0.24 mM and 0.17 mM) were obtained for nitrate and nitrite. Based on the derivatization with DAN, Wang et al. [34] employed CE and fluorescence detection to measure nitrite in human plasma, resulting in preferable linearity (R^2^ = 0.9975) between 2 nM and 500 nM, as well as an LOD of 0.6 nM in the initial plasma specimens.

Zhang et al. [65] constructed a novel capillary open tube column prepared with a methyl diethanolaminated nano latex. The column had a positive amino charge on the inner wall capable of effectively controlling EOF size and direction, changing the selectivity of the modified column, affecting the CE column efficiency, and acquiring relatively steady but opposite EOF values. An LOD of 1 ng/mL was obtained for both nitrite and nitrate.

Capillary ion analysis (CIA) is a new CE technique developed in the early 1990s. Taus et al. [66] utilized CIA coupled with UV–Vis detection to formulate a nitrite detection method targeting the blood, yielding a linearity ranging from 0.25 mM to 5 mM. The RSDs were 14.7%. Both nitrate and nitrite showed an LOD of 0.025 mmol/L. Detection was rapid with this method, but it may be affected by other ions as well as hemoglobin.

Notably, Siegel et al. [67] adopted electrochemical detection (ME-EC) together with microchip electrophoresis to detect nitrite in RAW 264.7 cells by applying a Pt-black working electrode with a polydimethylsiloxane (PDMS)/glass microchip fabrication. An LOD of 0.50 mM for nitrite was achieved. This method provided feasibility for future studies of NO in single cells and NO-related diseases. CE has no between-run residues, simple sample preparation and other unique merits in bio-analysis.

### 3.6. Paper-Based Analytical Devices

**Figure 11:**
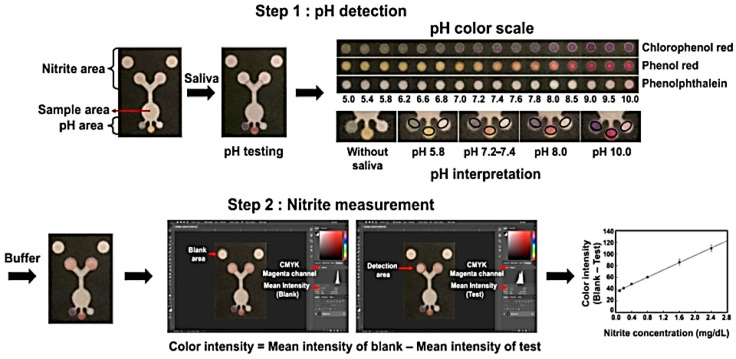
Image of the PAD and scheme for pH and nitrite determination. Step 1 describes the process for naked eye determination of the pH. The black circle indicates the position for pH measurement. The color inside the black circle is then compared to the pH color scale for pH determination. Step 2 describes the process for quantifying color intensity measuring using the program Photoshop^®^ (version 23) for nitrite concentration determination. The concentration of nitrite in saliva samples was calculated from the calibration curve. Reproduced from ref. [70].

In terms of biological samples containing nitrite and nitrate, the analysis, either spectroscopic method or chromatography as well as CE, requires specialized laboratory operators and instruments, which greatly limits remote monitoring. Therefore, identification of a method characterized by instant monitoring, low cost, and easy operation has become a research hotspot. Paper-based analytical devices (PADs) may provide point-of-care diagnostics with a potential solution [68,69,70,71,72,73,74,75,76,77,78,79], as summarized in Table 5. The schematic assembly of the PAD reported by Noiphung et al. [70] is shown in Figure 11.

Zhang et al. [71], utilizing electrokinetic stacking (ES) based on a colorimetric reaction on a PAD, achieved nitrite detection on a smartphone. The sensitivity can be improved by 160-fold for nitrite under optimum circumstances (LOD: 73 ng/mL (0.86 μM)). Using this method, high recovery (91.0~108.7%) and good reproducibility (RSD < 9%) were obtained.

PADs were used to measure nitrite concentration in artificial saliva by the Griess reaction for 30 min and absorbance measurement at 548 nm with a UV–Vis spectrometer [72]. Within the 0.1−2.4 mg/dL range, satisfactory linearity of nitrite was obtained.

**Figure 12:**
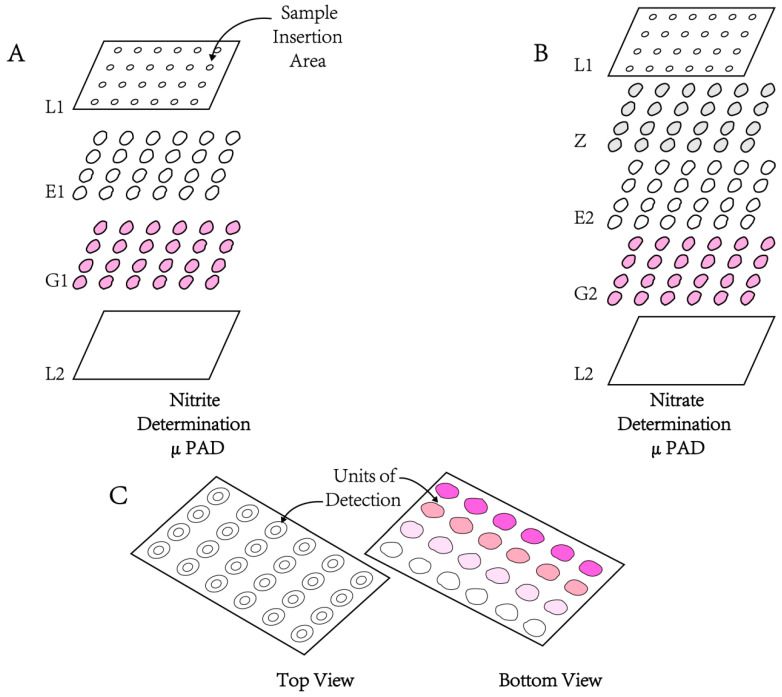
Schematic assembly of the μPAD for the nitrite (**A**) and nitrate (**B**) determination and the schematic representation of the device after sample placement; (**C**); L1, top layer of the laminating pouch; L2, bottom layer of the laminating pouch; E1, empty layer; G1, Griess reagent layer (5 μL per disc); Z, zinc embedded layer; E2, empty layer; G2, Griess reagent layer (10 μL per disc). Reproduced from ref. [74].

The advantages of PADs include low cost, rapid diagnosis, satisfactory response ranges, long stability and environmental friendliness [71]. As another direction of PADs and an emerging category of point-of-care devices, microfluidic PADs (µPADs) are able to integrate simple paper strip tests with the properties of conventional microfluidic devices [72,73,74,79]. The schematic assembly of the μPAD for the nitrite (A) and nitrate (b) reported by Ferreira et al. [74] is shown in Figure 12.

de Oliveira et al. [76] reported that the nitrite LODs were 2.34 µM and 4.35 µM in artificial urine samples using 2D and 3D devices. Ferreira et al. [77] reported a microfluidic technique with reference to the Griess reaction and reduction of nitrate to nitrite by NADPH reductase and the incubation time of this method was 20 min. Over the range from 0.14 mmol to 1 mmol, the linearity was preferable. An LOD of 40 µM, LOQ of 140 µM and RSD < 8% were obtained using this method.

Bhakta et al. [75] developed a μPAD fabricated with wax printing to detect nitrite by a colorimetric reaction associated with the Griess reaction. In the 10 μmol/L−1000 μmol/L range, visual determination of nitrite showed good linearity with an LOD of 10 µM.

Microfluidic chip technology is fast and can achieve on-site monitoring. However, the optimization of various conditions in the production of microfluidic chips is time- and labor-consuming, and a μPAD is mostly combined with a colorimetric method; thus, its sensitivity may not meet the needs for monitoring low concentrations in samples.

Gaikwad et al. [78] reported a fluorescence-based microfluidic method to improve the sensitivity of μPADs. In the range of 0 µM−20 µM, there was good linearity, and sensitivity was greatly improved (LOD: 60 nM). Therefore, μPADs can be combined with fluorescence assays, immunoassays and other techniques, in addition to colorimetric assays, to analyze biological samples.

### 3.7. Electrochemical Sensors

Various electrochemical sensors have been employed to measure nitrite, where neither complex devices nor specimen preparation procedures are required [80,81,82,83,84,85,86,87,88,89,90,91]. We summarize these electrochemical sensors in Table 6. Considering the rapid response, selectivity, simplicity, repeatability, sensitivity and the advantages involving simple operation, chemically modified electrodes (CMEs) are increasingly used to detect nitrite content in biological samples. In recent years, nano materials as electrochemical sensors have attracted great interest in this field [80,81,82,83,84,85,86,87]. In addition, as a robust, speedy, precise and calibration-free method for ion detection in water specimens developed from ion selective membranes, thin layer coulometric detection has attracted considerable attention. Cardoso et al. [88] fabricated a custom-made coulometric cell for nitrite detection, which was composed of a counter (CE) and working electrode (WE) made of Ag/AgCl. The LOD of this method was 10 μM.

Caetano et al. [89] reported that a screen-printed electrode (SPE)/MWCNT could be combined with batch-injection amperometric (BIA) for detection of nitrite. Good linearity was obtained for nitrite over the 1 mmol/L−500 mmol/L range. The LOD of this method was 60 μM. Moreover, batch injection analysis with multiple-pulse amperometric (BIA-MPA) detection had high accuracy (RSD < 1.3%) and speed (160/h) in addition to being free from sample–matrix interferences.

In particular, Gross et al. [90] applied a dual-plate microtrench (inter-electrode gap at approximately 15 µm) made of gold-coated glass to a smart electrode. This method is simpler than the use of complex modified electrodes and is sensitive (LOD of 24 μM).

Cardoso et al. [91] developed a method for measuring nitrite in urine and saliva using 3D-printed graphene-polylactic acid (PLA) electrodes, which were manufactured using 3D printing technology and could be mass produced. Satisfactory results were obtained, with recoveries of 70–110%, an LOD of 0.03 μmol, and favorable linearity within 0.5–250 μmol.

Electrochemical sensors, particularly those based on nanomaterials, have remarkable selectivity; they do not require complicated sample pretreatment and can be used for detection in serum, urine, and saliva after direct dilution. Although they have many advantages (e.g., high sensitivity and no tedious pretreatment), their disadvantage is the complex modification of electrodes.

### 3.8. GC-MS

GC-MS has become an alternative of interest due to its superior separating efficiency and outstanding selectivity along with preferable sensitivity. GC-MS requires derivatization under general conditions.

Urine nitrite level was determined using GC-MS after NAT derivatization by Liu et al. [17]. The method was sensitive (LOD: 1 ng/mL) with good repeatability (RSD: 3.19~4.06%).

**Figure 13:**
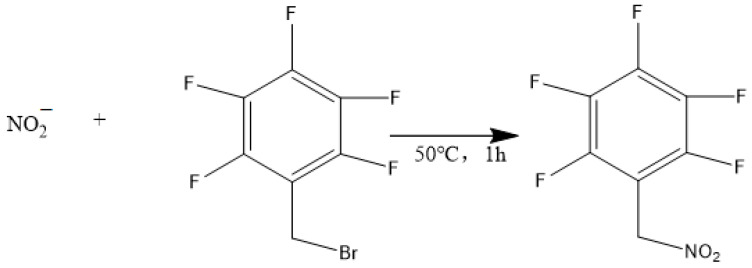
The PFB-Br scheme derivatization for nitrite.

The GC-MS method usually requires PFB-Br derivatization [14,15,58,92,93,94,95], and has good sensitivity and specificity. Its disadvantage is the long derivation time (50 °C for 60 min). The reaction equation is shown in Figure 13.

Hanff et al. [15] employed GC-NCI-MS plus PFB-Br to detect nitrite and nitrate content in human urine and plasma. The LODs of nitrite and nitrate were 0.2 fmol and 4.8 fmol, respectively.

Ata et al. [58] obtained the LOQs and LODs for nitrite (0.04 ng/L and 0.01 ng/L) and nitrate (0.07 ng/L and 0.02 ng/L) via GC-MS combined with PFB-Br derivatization. Compared with HPLC-FD, GC-MS had a higher sensitivity.

Schwarz [16] utilized PFB-Br combined with GC-MS to quantify nitrite in human plasma, erythrocytes, and whole blood with the N15 isotope as the IS (during quantitative measurement, stable isotope-marked anions serve as ideal ISs). The reaction products in this method were stable, sensitive and accurate. However, the reaction time was long and pretreatment was complex. Moreover, LLE was usually used to extract the reaction products. Using toluene and ethyl acetate as the extraction solvents is potentially harmful to the experimenters. Another disadvantage is that the nitrate derivatization product was unstable, resulting in unsatisfactory results for nitrate determination [94,95,96].

### 3.9. Summary

Although all these techniques have been developed, including spectrophotometry, chemiluminescence, fluorometric, IR, Raman emission, GC-MS, HPLC, and HPLC-MS/MS, the traditional spectrophotometric and colorimetric methods have low sensitivity and susceptibility to matrix interferences. In the case of HPLC, it requires complicated sample pretreatment in order to have high sensitivity and eliminate the matrix interferences; for GC-MS, derivatization is required [9]. Extensive application of CE as a substitution assay for biological specimens has been accomplished due to its merits (e.g., low running cost, strong resolving power, short sample preparation time, and high efficiency) [4,9,10,63]. However, the costly instruments need to be manipulated by professionals and/or complicated specimen preparation techniques for these methods prevent their application in rural and remote regions urgently requiring environmental monitoring of nitrites. The pursuit of rapid testing and portable analytical instruments yielded electrochemical sensors and PAD/μPAD. Electrochemical sensors have sensitivity and simplicity but require modification of the electrode to achieve high sensitivity. PAD/μPAD is fast, can achieve field monitoring, but its sensitivity should be improved and its fabrication is tedious. Overall, it is still challenging for analysts to develop an ideal analytical technique, which is simple, sensitive, specific and portable, to examine nitrite and nitrate content in biological samples.

## 4. Conclusions

The analytical methods used in the recent decade for nitrite and nitrate detection in biological samples, covering both preprocessing and measuring methods, are comprehensively reviewed in this article. In contrast to previous reviews, the present review emphasizes novel solvents (deep eutectic solvents, ionic liquids, etc.), paper-based analytical devices, materials (polymers, nanomaterials and so on), and electrochemical sensors, all of which are used to extract, separate and measure nitrite and nitrate from biological samples. There are adequate simple pretreatment methods (e.g., ultrafiltration, protein precipitation and dilution) due to the fairly clean sample matrices and simple composition (e.g., urine and saliva). Nevertheless, sample preprocessing with higher complexity is still challenged by matrix effects and sample loss. Hence, it is necessary to identify a larger number of efficient and advanced pretreatment methods such as CPE, IL-DLLME and DESs-DLLME. And we believe that DLLME has a very broad application prospect because of its rapidity, simplicity, low cost, high enrichment factor, negligible consumption of extraction solvents, and environmental benignity.

Until now, spectroscopic methods were the mainstream approaches for nitrite and nitrate measurement in recent years, with the merits of simple operation and low equipment cost. However, their demerits include low sensitivity, high detection limit and interference issues. IC and CE only provide a retention time for nitrate and nitrite identification, which results in limited specificity. Moreover, CE is not a routine technique for anion test identification and has limitations for complex samples. Although LC coupled with UV has low sensitivity and requires complex pretreatment processes, fluorescence detection or mass spectrometry conjugated with LC is feasible to obtain high sensitivity and repeatability. GC-MS on the basis of derivatization with PFB-Br can accurately analyze biological samples for nitrite and nitrate concentrations simultaneously and quantify them by adding a stable isotope-labeled internal standard, which can effectively avoid experimental errors and greatly improve quantification accuracy. The development of rapid, economical and green methods with high sensitivity and throughput for the assay of nitrite and nitrate is expected to be the mainstream direction of research in the future. As far as we are concerned, paper-based analytical devices are worth popularizing because of their instant monitoring, low cost, easy operation, satisfactory response ranges, long stability and environmental friendliness.

## Figures and Tables

**Table 1:** The summary of the pretreatment of nitrite and nitrate in biological samples.

Pretreatment Methods	Derivatization Reagents	Analytical Technique	Analyte	Matrix	Recoveries	Precision	Ref.
Centrifugation and dilution	AHNDMS	Spectrophotometry	nitrite	saliva	98%	<0.82%	[12]
Centrifugation/Filtration	griess	Spectrofluorimetry	nitrite	water, sausage, soil	95–108%	<2.88%	[13]
Centrifugation/LLE	PFB-Br	GC-MS	nitrite nitrate	blood	92–99%	<16.1%	[16]
Protein precipitation	PFB-Br	GC-MS	nitrite nitrate	erythrocytes, plasma	95–113%	0.2–16.2%	[16]
Centrifugation/ ultrafiltration	glutathione	LC-MS/MS	nitrite	plasma	92–108%	<4.4%	[30]
Protein precipitation	griess	HPLC-UV	nitrite nitrate	plasma	n.d.	n.d.	[31]
Protein precipitation	/	IC-ED	nitrite	blood	56–72%	<14.9%	[32]
Protein precipitation	NO	CE- fluorescence	nitrite nitrate	plasma	92–113%	<2.6%	[33]
Protein precipitation	DAN	CE- fluorescence	nitrite	plasma	85–112%	<1.1%	[34]
Protein precipitation	arsenate	Spectrophotometry	nitrite nitrate	blood	99–101%	<2.1%	[35]
Protein precipitation/ATPE	OPE	Spectrofluorimetry	nitrite	urine	93–105%	<1.7%	[28]
Protein precipitation	I3	Chemiluminescence	nitrite	blood	n.d.	n.d.	[36]
Dilution/online SPE	DAN	LC-MS/MS	nitrite nitrate	urine	99–112%	<7.4%	[26]
Soak, vortex, ultrasonic, centrifugation/online SPE	DAN	LC-MS/MS	nitrite nitrate	feces	99–112%	<7.4%	[26]
LLE	PFB-Br	GC-MS	nitrite nitrate	urine	n.d.	n.d.	[14]
LLE	PFB-Br	GC-MS	nitrite nitrate	urine	91–113%	0.92–19%	[15]
LLE	DAN	GC-MS	nitrite	urine	90–113%	<5.16%	[17]
LLE	griess	HPLC-UV	nitrite nitrate	human plasma	98–102%	<8.77%	[37]
CPE	griess	HPLC-DAD	nitrite nitrate	plasma, urine	90–98%	<4.19%	[18]
CPE	griess	spectrophotometry	nitrite	urine, blood	92–101%	n.d.	[19]
CPE	griess	spectrophotometry	nitrite	meat, water	91–103%	<3.4%	[20]
HS-SDME	griess	pectrophotometry	Nitrite	water	98–137%	<10.6%	[23]
IL-DLLME	griess	HPLC-UV	nitrite	water and saliva	96–107%	4.1%	[24]
VA-DLLME	griess	HPLC-UV	nitrite	saliva	90–115%	<4.6%	[25]
EME	/	IC-CD	nitrite	amniotic fluids	n.d.	<11%	[29]
SPME	sodium cyclamate	μPD-OES	nitrite	simulated gastric content, serum	96–103%	4.1%	[27]

Note: AHNDMS: 4-amino-5-hydroxynaphthalene-2,7-disulphonic acid monosodium salt; ATPE: Aqueous two-phase extraction; CPE: cloud point extraction; DAN: 2,3-diaminonaphthalene; DLLME: dispersive liquid-liquid microextraction; EME: Electromembrane extraction; HPLC-CD: High-performance liquid chromatography–conductivity detector; HPLC-DAD: High-performance liquid chromatography–diode array detection; HS-SDME: headspace single-drop liquid phase microextraction; IC: Ion chromatography; IC-ED: Ion chromatography–electrochemical detector; IL-DLLME: Ionic liquid–dispersive liquid–liquid microextraction; OPD: o-phenylenediamine; SPME: solid phase micro extraction; μPD-OES: point discharge optical emission spectrometry; VA-DLLME: vortex-assisted dispersive liquid–liquid microextraction. n.d. means not found.

**Table 2:** Comparation of the advantages and disadvantages for different pretreatment technologies.

LLE	LPME	SPE	SPME	Simple Sample Treatment
**Evolution**				
CPE, ATPE	HS-SDME, DLLME, EME	On-line SPE	HS-SPME	Protein precipitation, dilute and shoot
**Advantages**				
Being reliable	Good purification effect	Less reagent consumption than LLE	High enrichment factor	Low cost
Wide extraction range	High enrichment and extraction efficiency	No emulsification occurs during treatment	Each extraction head can be used more than 50 times	Time-saving
Simple equipment and easy to operate	Less organic solvent at the level of a microliter	Simplified sample handing process	Different adsorption fibers meet a variety of application requirements	Easy accessibility
Mild extraction conditions	Easy to automate with a low cost	High selectivity and reproducibility due to the use of highly efficient and selective adsorbents	Used with automatic sampler to ensure the accuracy of the experiment	Appropriate for simple and clean samples
**Disadvantages**				
Easily emulsifying and poor selectivity	The extraction efficiency is susceptible	Higher cost than LLE	Carry-over problems caused by repeated analysis with the same fiber	Limited ability to handle complex samples
Large amount of organic solvent				Limited potential for automation and efficiency

**Table 3:** The information of nitrite and nitrate detected by spectrophotometry, spectrofluorometry and colorimetry.

Methods	Matrix	Reagents	λmax (nm)	Linearity Range	Detection Limit (ng/mL)	Reaction Time (min)	Ref.
spectrophotometry	saliva samples	AHNDMS	560	0.1 μg/mL–1.6 μg/mL	7.5	2	[12]
spectrophotometry	urine, blood	griess	490	10 ng/mL–400 ng/mL	2.5	n.d.	[19]
spectrophotometry	blood samples	arsenomolybdneum blue	840	2 μg/mL–10 μg/mL	3	30	[37]
spectrophotometry	urine	[Ru(npy)([9]aneS_3_)(CO)](ClO_4_)	483	1 µM–840 µM	26.91	1	[44]
spectrofluorometry	urine	BSA-Au NCs	660	1 μM–100 μM	3.45	60	[46]
spectrofluorometry	urine	GQDs	480	0.025 μg/mL–0.09 μg/mL	0.373	5	[47]
spectrofluorometry	urine	NIR-CDs	675	1 μM–50 μM	3.864	n.d.	[48]
spectrofluorometry	urine	hydroxypropyl-β-cyclodextrin	568	5 ng/mL–1000 ng/mL	1.5	n.d.	[28]
colorimetry	artificial urine	griess	n.d.	0.78 μM–200 μM	110.4	1	[51]
colorimetry	urine	TMB	452	0.5 μM–30 μM	6.9	1	[55]

Note: n.d. means not found.

**Table 4:** HPLC coupled with different detectors for determination of nitrite and nitrate in biological samples since 2010.

Matrix	Analytes	Column	Mobile Phase	Detector	Preparation	Linear Range	LOD	LOQ	Ref.
blood urine	nitrite nitrate	C18 column (150 mm × 4.6 mm, 5 mm)	A: acetonitrile B: methanol C: 1%tetrabutylammonium hydroxide. gradient elution	UV	decolorization and protein precipitation, CPE	10–1000 ng/mL (nitrite) 0.1–10 μg/mL (nitrate)	1 ng/mL for nitrite; 0.1 μg/mL for nitrate	n.d.	[18]
saliva	nitrite	VP-ODS column (150 mm × 4.6 mm, 5 μm)	methanol/water (90:10, *v*/*v*) isocratic elution	UV	IL-DLLME	0.4–500.0 μg/L	0.05 μg/L	0.4 μg/L	[24]
urine and saliva	nitrite	C18 column (250 mm × 4.6 mm, 5 μm)	95% methanol and 5% water isocratic elution	UV	vortex-assisted dispersive liquid–liquid microextraction	1–300 μg/L	0.2 μg/L	1 μg/L	[25]
rat serum	nitrite nitrate	XBridge C18 (2.1 mm × 50 mm, 3.5 µm)	15% (*v*/*v*) acetonitrile in 20 mM sodium phosphate buffer (pH 10) isocratic elution	FD	centrifugation	0.02–2.00 μM for nitrite: 0.3125–20 μM for nitrate	0.003 μM for nitrite 0.083 mM for nitrate	0.009 μM for nitrite; 0.250 μM for nitrate	[38]
human plasma	nitrite nitrate	120 CN (25 cm × 0.46 cm, 5 μm)	methanol and water (57.5:42.5, *V*:*V*) isocratic elution	UV	pre-column derivatization of nitrite anion using the Griess; nitrate with vanadium chloride (III)	0.1–50 μM (nitrite) 1–500 μM (nitrate)	n.d.	0.1 μM for nitrite	[37]
serum	nitrite nitrate	POLAR-RP column (250 mm × 4.6 mm, 4 µm)	A: acetonitrile B: double distilled–deionised water consisted of 0.1% trifluoroacetic acid gradient elution	FD	derivatization then LLE	1–5000 ng/L (nitrite) 1–100 μg/L (nitrate)	0.13 ng/L for nitrite; 0.19 ng/L for nitrate	0.43 ng/L for nitrite; 0.58 ng/L for nitrate	[58]
human saliva	nitrite nitrate	Phosphatidylcholine Column (4.6 mm × 150 mm, 10 µm)	20 mM NaCl isocratic elution	DAD	dilution and centrifugation	8.98–18.52 µg/mL (nitrate) 3.50–5.34 µg/mL (nitrate)	4.56 ng/mL for nitrate; 4.21 ng/mL for nitrite	15.21ng/mL for nitrate; 14.03 ng/mL for nitrite	[59]
rabbit blood	nitrite nitrate	X Bridge C18 (2.1 mm × 50 mm, 2.5 μm)	A: tetrabutylammonium hydroxide 5 mM brought to pH 2.5 with sulfuric acid B: acetonitrile C: methanol gradient elution	UV	pre-column derivatization of nitrite anion using the Griess	6–400 μg/L (nitrite) 0.2–200 μg/mL (nitrate)	0.06 μg/mL for nitrate	2 ng/mL for nitrite and 200 ng/mL for nitrate	[31]
rat plasma	nitrite nitrate	An Acquity UPLC^®^ BEH C18 column (2.1 mm × 50 mm, 1.7 μm)	A: tetrabutylammonium hydroxide (12 mM), potassium dihydrogen phosphate (pH 7.0; 10 mM) B: consisted of tetrabutylammonium hydroxide (2.8 mM), methanol (30% *v*/*v*), potassium dihydrogen phosphate (pH 5.5; 100 mM). gradient elution	PDA	using filter plate to deprotein	4–500 μM (nitrite) 6–400 μM (nitrate)	n.d.	4 μM for nitrite; 6 μM for nitrate	[60]
rat plasma/urine	nitrite nitrate	C18 (250 mm × 4.6 mm, 5 µm)	methanol–water (containing 0.60 mM of phosphate salt and 2.5 mM TBAP) (2:98, *V*:*V*) isocratic elution	PDA	deproteinization with acetonitrile	1–800 μM for nitrate and nitrite	0.075 μM for nitrate	0.25 μM for nitrate and nitrite	[61]

Note: n.d.: not found; LOD: limit of detection; LOQ: limit of quantification; PDA: photo-diode array; FD: fluorescence detection; DAD: diode array detector; IC: ion chromatography; TBAP: tetrabutylammonium perchlorate.

**Table 5:** Summary of paper-based analytical methods for determination of nitrite and nitrate in biological samples.

Matrix	Analytes	Paper Material	Fabrication Technique	Derivatization	Analytical Range	LOD	LOQ	Ref.
artificial saliva	nitrite	Whatman filter paper	a wax printing	Griess	0.1–2.4 mg/dL	n.d.	n.d.	[70]
saliva	nitrite	glass fiber	electrokinetically stacking	Griess	0.075–1.0 μg/mL	73 ng/mL	n.d.	[71]
saliva	nitrite	Whatman filter paper	the laminating pouches were passed through the laminator	Griess	5–250 μM	0.05 μM	0.17 μM	[74]
saliva	nitrate	Whatman filter paper	the laminating pouches were passed through the laminator	Griess	0.2–1.2 mM	0.08 mM	0.27 mM	[74]
saliva	nitrite	cellulose filter paper	a wax printing	Griess	1–100 μM	10 μM	n.d.	[75]
urine sample	nitrite	cellulose filter paper	an inexpensive home cutter printer and plastic adhesives	Griess	5–100 μM	2.34 μM	n.d.	[76]
blood serum	nitrite	cellulose filter paper	an inexpensive home cutter printer and plastic adhesives	Griess	5–600 μM	4.35 μM	n.d.	[76]
human urine samples	nitrite	Whatman filter paper	the laminating pouches were passed through the laminator	Griess	0.14–1.0 mM	0.04 mM	0.14 mM	[77]
blood plasma	nitrite	silicon substrate	an acoustics-based plasma separation device	Griess	0–20 µM	60 nM	n.d.	[78]

**Table 6:** Electrochemical sensor for determination of nitrite and nitrate in biological samples since 2010.

Electrode	Modification	Method	Analytes	Linearity Range	LOD (nM)	Precision	Recovery	Ref.
Pt	NaR–SOD1–CNT–PPy–Pt	CV	nitrite	100 nM–1 mM	50 nM	3.57%	n.d.	[22]
Pt	NaR–SOD1–CNT–PPy–Pt	CV	nitrate	500 nM–10 mM	200 nM	2.98%	n.d.	[22]
Pt	SOD1–CNT–PPy–Pt	CV	nitrite	0.5–2000 μM	0.5 ± 0.025 μM	n.d.	n.d.	[80]
GCE	CDP–GS–MWCNTs	CV	nitrite	5 µM–6.75 mM	1.65 μM	<3.7%	95.0% and 106.6%	[81]
GCE	nano-Au/p-TA	DPV	nitrite	15.9–277.0 µM	0.89 μM	<2.3%	91.0% and 109.0%	[82]
GCE	La–MWCNTs	CA	nitrite	0.40–0.71 mM	103 nM	<4.2%	101.6% and 101.7%	[83]
GCE	Fe(III)P/MWCNTs	CV	nitrite	1.00 μM–1.6 mM	0.50 μM	<4.5%	98.0% and 110.0%	[84]
GCE	CTAB-GO/MWNT	DPV	nitrite	5.0–800 μM	1.5 µm	2.50%	99% and 105%	[85]
ZnO-screen printed electrodes	CuCP	CV	nitrite	100 nM–1 mM	100 nM	n.d.	n.d.	[86]
GCE	Fe_3_O_4_@Au@Cys/rGO	CV	nitrite	0.03–344 and 344–2215 µM	8 nm	<3.26%	98.5–104%	[87]
Ag/AgCl wire	A cobalt(II) tert-butyl salophen compound	CA	nitrite	20–100 µM	10 µm	<1%	n.d.	[88]
SPCE	MWCNT	BIA-MPA	nitrite	1–40 µM	0.3 µM	<1.3%	77–121%	[89]
gold-gold microtrench electrode	silver	CA	nitrate	200–1400 μM	24 μM	<6.9%	108%	[90]
3D-printed electrode	graphene/PLA	BIA-MPA	nitrite	0.5–250 µM	0.03 µML	1.10%	70 and 110%.	[91]

Note: GCE: glassy carbon electrode; SPCE: screen printed carbon electrode; nano-Au/p-TA: au-nanoclusters on poly(3-amino-5-mercapto-1,2,4-triazole); CDP–GS–MWCNTs: CD cross-linked pre-CD–graphene sheet–multiwall carbon nanotubes; Fe(III)P/MWCNTs: non-covalent iron(III)-porphyrin-MWCNTs; La–MWCNTs: lanthanum–multiwalled carbon nanotube nanocomposites; NaR–SOD1–CNT–PPy–Pt: copper, zinc superoxide dismutase (SOD1) and nitrate reductase (NaR) coimmobilized on carbon nanotubes(CNT)–polypyrrole (PPy) nanocomposite modifified platinum electrode; CTAB-GO/MWNT: hexadecyl trimethyl ammonium bromide (CTAB) functionalized graphene oxide (GO)/multiwalled carbon nanotubes; CuCP: copper(II) chlorophyllin; PLA: polylactic acid; Fe_3_O_4_@Au@Cys: Fe_3_O_4_@Au core-shell nanoparticles; rGO: reduced graphene oxide; CV: Cyclic voltammetric; CA: Chronoamperometric; DPV: Differential pulse voltammograms; BIA-MPA: Batch injection analysis with multiple-pulse amperometric. n.d. means not found.

## Data Availability

The article only utilizes available data from this study.

## Abbreviations

AHNDMS	4-amino-5-hydroxynaphthalene-2,7-disulphonic acid monosodium salt
4-ATP	4-aminothiophenol
ATPE	aqueous two-phase extraction
BGE	back ground electrolyte
BIAMPA	batch injection analysis with multiple-pulse amperometric
BPS	bathophenanthroline disulfonic acid
BSA-Au NCs	bovine serum albumin stabilized gold nanoclusters
CIA	capillary ion analysis
CME	chemically modified electrode
CNT	carbon nanotube
CPE	cloud point extraction
CE	capillary electrophoresis
CZE	capillary zone electrophoresis
DAD	diode array detection
DLLME	dispersive liquid–liquid microextraction
DESs	deep eutectic solvents
DAN	2,3-diaminonaphthalene
ES	electrokinetic stacking
EF	enrichment factor
ER	extraction recovery
EOF	electroosmotic flow
FD	fluorescence detection
FIA	flow injection analysis
GC-MS	gas chromatography-mass spectrometry
GC-NCI-MS	gas chromatography-negative chemical ionization-mass spectrometry
GQDs	graphene quantum dots
HP-β-CD	hydroxypropyl-β-cyclo-dextrin
HPLC	high performance liquid chromatography
HS-SDME	headspace Single drop liquid phase microextraction
IC	ion chromatography
ILs	ionic liquids
LLE	liquid–liquid extraction
LPME	liquid phase microextraction
LC-MS/MS	liquid chromatography-tandem mass spectrometry
LOD	limit of detection
LOQ	limit of quantification
ME-EC	microchip electrophoresis method with electrochemical detection
MNP_S_	magnetic nanoparticles
NAT	2,3-naphthotriazole
NCs	nanoclusters
NIR-CDs	near-infrared carbon dots
NO_2_^−^	nitrite
NO_3_^−^	nitrate
NO	nitric oxide
PADS	paper-based analytical devices
PDA	photo-diode array
PDMS	polydimethylsiloxane
PLA	polylactic acid
SIA	sequential injection analysis
SPE	solid phase extraction
SERS	surface enhancement Raman spectroscopy
TMB	3,3′,5,5′-tetramethylbenzidine
µPADs	microfluidic paper-based analytical devices
UPLC	ultra performance liquid chromatography
[Ru(npy)([9]aneS_3_)(CO)](ClO_4_)	(Ru = Ruthenium; npy = 2-(1-naphthyl)pyridine, [9]aneS_3_ = 1,4,7-trithiacyclononane)
